# Fusion Cytokines IL-7-Linker-IL-15 Promote Mycobacterium Tuberculosis Subunit Vaccine to Induce Central Memory like T Cell-Mediated Immunity

**DOI:** 10.3390/vaccines8040715

**Published:** 2020-12-01

**Authors:** Chunxiang Bai, Lijun Zhou, Junxia Tang, Juanjuan He, Jiangyuan Han, Hongxia Niu, Bingdong Zhu

**Affiliations:** 1 Gansu Provincial Key Laboratory of Evidence Based Medicine and Clinical Translation & Lanzhou Center for Tuberculosis Research, School of Basic Medical Sciences, Lanzhou University, Lanzhou 730000, China; baichx13@lzu.edu.cn (C.B.); hejj2014@lzu.edu.cn (J.H.); hanjy17@lzu.edu.cn (J.H.); niuhx@lzu.edu.cn (H.N.); 2 Institute of Pathogen Biology, School of Basic Medical Sciences, Lanzhou University, Lanzhou 730000, China; baichx15@lzu.edu.cn (L.Z.); tangjx2016@lzu.edu.cn (J.T.)

**Keywords:** M. tuberculosis, subunit vaccine, fusion cytokines, IL-7-Linker-IL-15, central memory like T cells

## Abstract

Tuberculosis (TB), caused by *Mycobacterium tuberculosis* (*M. tuberculosis*), is among the most serious infectious diseases worldwide. Adjuvanted protein subunit vaccines have been demonstrated as a kind of promising novel vaccine. This study proposed to investigate whether cytokines interliukine-7 (IL-7) and interliukine-15 (IL-15) help TB subunit vaccines induce long-term cell-mediated immune responses, which are required for vaccination against TB. In this study, mice were immunized with the *M. tuberculosis* protein subunit vaccines combined with adnovirus-mediated cytokines IL-7, IL-15, IL-7-IL-15, and IL-7-Linker-IL-15 at 0, 2, and 4 weeks, respectively. Twenty weeks after the last immunization, the long-term immune responses, especially the central memory-like T cells (T_CM_ like cell)-mediated immune responses, were determined with the methods of cultured IFN-γ-ELISPOT, expanded secondary immune responses, cell proliferation, and protective efficacy against *Mycobacterium bovis* Bacilli Calmette-Guerin (BCG) challenge, etc. The results showed that the group of vaccine + rAd-IL-7-Linker-IL-15 induced a stronger long-term antigen-specific T_CM_ like cells-mediated immune responses and had higher protective efficacy against BCG challenge than the vaccine + rAd-vector control group, the vaccine + rAd-IL-7 and the vaccine + rAd-IL-15 groups. This study indicated that rAd-IL-7-Linker-IL-15 improved the TB subunit vaccine’s efficacy by augmenting T_CM_ like cells and provided long-term protective efficacy against *Mycobacteria*.

## 1. Introduction

Tuberculosis (TB), mainly caused by *Mycobacterium tuberculosis* (*M. tuberculosis*), ranks as the first leading cause of death from a single infectious disease worldwide [1]. The effective way to prevent and control the infectious disease is vaccination. A potentially successful *M. tuberculosis* vaccine should have the ability to induce more long-term antigen-specific immune memory cells, which could expand rapidly when it encounters the same pathogen. Currently, attenuated *Mycobacterium bovis* Bacilli Calmette-Guerin (BCG) is the only approved TB vaccine in clinic. However, it provides variable protection against TB [2,3]. There are reports that BCG vaccination mainly induces shorter-term effector memory T cells (T_EM_) rather than long-lived central memory T cells (T_CM_) [4,5]. Thus, it is urgently required to develop novel vaccines for inducing enough T_CM_ to produce long-term protection against *M. tuberculosis* [6].

The protein subunit vaccine is a kind of promising TB vaccine. So far, there are at least 14 TB vaccines in clinical trials, including four protein subunit vaccines, such as ID93 + GLA-SE, H1/H56 + IC31, H4 + IC31, and M72 + AS01E [7]. Protein subunit vaccines require an adjuvant to induce a stronger immune response. Adjuvants can have an effect on the strength and duration of immune responses [8]. We have developed an adjuvant composed of N,N’-dimethyl-N,N’-dioctadecylammonium bromide (DDA), and polyinosinic-polycytidylic acid [Poly (I:C)] (DP for short), which could help the TB subunit vaccine induce strong Th1-type cell-mediated immunity [9]. Considering that an effective TB vaccine is needed to induce long-term immune memory mediated by T_CM_ rather than T_EM_ [10,11], there is also need to survey how to promote the development of T_CM_ during vaccination.

IL-7 and IL-15 are both members of the γ-chain cytokine family, which share γ-chains of IL-2R for signal transduction and play a role in immune memory. Some studies showed that IL-7 and IL-15 were essential for development and maintenance of memory T cells [12,13,14]. IL-7 could regulate T cell homeostasis and enhance the survival of memory T cells [15,16]. While IL-15 could promote the differentiation of CD8^+^ memory T cells [17]. IL-7 and IL-15 had been proved to enhance the immune memory induced by many vaccines, such as vaccines against Toxoplasma gondii [18,19], HIV-1 vaccine [20], and BCG against *M. tuberculosis* [21]. However, it is still uncertain if both IL-7 and IL-15 promote the development of T_CM_ and enhance the protective efficacy of the vaccines. For example, it was reported that the administration of IL-15 increased antigen-specific CD8^+^ memory T cells after BCG infection, but the protective efficacy against *M. tuberculosis* was not improved [22]. Thus, it is necessary to explore the immune memory character induced by IL-7 and IL-15 combined with vaccines. In our study, we co-administrated *M. tuberculosis* subunit vaccine ESAT6-Ag85B-MPT64<190-198>-Mtb8.4-Rv2626c (LT70 for short) [23] and Mtb10.4-HspX (MH for short) [24] in DP adjuvant, which showed stronger protective efficacy in mice, with rAd-IL-7, rAd-IL-15, rAd-IL-7-IL-15 and rAd-IL-7-Linker-IL-15 to investigate the properties of T cell immune.

## 2. Materials and Methods

### 2.1. Ethics Statement

Mice procedures were performed in accordance with the guidelines of China Council on Animal Care and Use. Animal license numbers SCXK (Gan) 2018-0002. The experiments were performed under isoflurane anesthesia to be made to minimize suffering.

### 2.2. Mice

Briefly, female 6–8-week old C57BL/6 mice were obtained from animal center of Lanzhou University (Lanzhou, China). All mice were maintained in special pathogen-free conditions in Lanzhou University and received free access to water and food throughout the study.

### 2.3. Preparation of Subunit Vaccines and Single Mycobacterial Antigens

The fusion proteins LT70 [23] and MH [24] were purified as previously described. Briefly, the plasmid encoding LT70 and MH were cloned into pET30(+) expression vector, respectively. Then, they were transformed into the Escherichia coli BL21 strain (DE3) for fusion proteins LT70 and MH from supernatant. Single mycobacterial proteins ESAT6, Ag85B, Rv2626c, and HspX were produced by Ni-NTA His column (Novagen) as previously indicated [25]. The purified LT70 and MH (10 μg/dose, respectively) were emulsified in adjuvant of DDA (250 μg/dose) and [Poly (I:C)] (50 μg/dose) with PBS in a volume of 200 μL for protein subunit vaccine (vaccine for short).

### 2.4. Construction of rAd-IL-7, rAd-IL-15, rAd-IL-7-IL-15, rAd-IL-7-Linker-IL-15 and rAd-Vector

The mouse IL-7 and IL-15 gene sequences were sub-cloned into shuttle plasmid pDC316, respectively. Subsequently, pDC316, pDC316-IL-7, pDC316-IL-15, pDC316-IL-7-IL-15, and pDC316-IL-7-Linker-IL-15, which the linker (Gly-Gly-Gly-Ser)_3_ [26] was connected between IL-7 and IL-15, combined with pBHGlox△E1, 3Cre adenovirus expression vector co-transfected into HEK293 cells by homologous recombination. The recombinant viruses of rAd-IL-7, rAd-IL-15, rAd-IL-7-IL-15, rAd-IL-7-Linker-IL-15, and the recombined adenoviral vector (rAd-vector), were verified with PCR analysis and cytopathic effect (CPE). The amplification of the recombinant virus was done in HEK293 cells. Adenoviral titers were determined as previously described [27].

### 2.5. Vaccine Immunization

Mice were divided into eight groups: The non-vaccinated mice received PBS; The vaccinated mice were topically immunized with vaccine, either co-administration of rAd-vector, rAd-IL-7, rAd-IL-15, rAd-IL-7-IL-15, rAd-IL-7-Linker-IL-15 or rAd-IL-7 + rAd-IL-15, respectively. For the group of PBS, mice were inoculated with PBS in a total volume of 200 μL/dose subcutaneously once at 0 week. For the other groups, vaccinations were performed at weeks 0, 2 and 4, respectively. Mice were immunized by the vaccine subcutaneously in a total volume of 200 μL/dose/mice on one side of the groin. At 30 min after protein vaccine immunization, 5 × 10^6^ PFU/100 μL/mice of rAd-vector, rAd-IL-7, rAd-IL-15, rAd-IL-7-IL-15, rAd-IL-7-Linker-IL-15, or rAd-IL-7 + rAd-IL-15, respectively were injected at the same site. 

### 2.6. Cultured IFN-γ ELISPOT assay In Vitro

Cultured IFN-γ ELISPOT assay was done as described previously [28]. Briefly, firstly lymphocytes (2 × 10^6^ cells/mL/well) were stimulated with a cocktail of ESAT-6, Ag85B, Rv2626c and HspX (2 μg/mL of each protein) in a 24-well plate. Media was replaced containing 100 U/mL IL-2 at days 3 and 7. At day 9, cultured lymphocytes were harvested. Then, cultured cells (1 × 10^6^ cultured cells/well) in the presence of additional antigen presenting cells (APCs) isolated freshly from C57BL/6 mice spleen at the ratio of 10:1 were restimulated with the same antigens and incubated in anti-mouse IFN-γ capture-mAb coated 96-well ELISPOT tech Company Limited, Shenzhen, China) either a cocktail of ESAT-6, Ag85B, Rv2626c, PPD or medium alone for an additional 20 h in the standard ELISPOT assay as the manufacturer’s protocols [28].

### 2.7. EdU Incorporation and Proliferation Assay

Lymphocytes (5 × 10^6^ cells/well) were stimulated with mixed antigens of ESAT-6, Ag85B, Rv2626c and HspX (2 μg/mL of each protein) in 24-well plates and at days 3 after antigen stimulation with EdU at a final concentration of 30 μM were added to the cells for another 4 days. At days 7, cells were harvested and treated with Click-iT reaction buffer (Cat. no. C10425, Click-iT™ EdU Flow Cytometry Assay Kit, Invitrogen™, Carlsbad, CA, USA) according to the manufacturer’s instructions. Then, cells were stained with anti-CD4-PE (RM4-5, eBioscience, San Diego, CA, USA) and anti-CD8-APC (53-6.7, BD, USA) [28]. Finally, samples were detected by flow cytometry.

### 2.8. IFN-γ Secretion Following Twice-Stimulation with Antigens

IFN-γ secretion following twice-stimulation with antigens in vivo and in vitro sequentially was performed [28]. Firstly, mice were stimulated with BCG (Danish 1331, 1 × 10^6^ CFU/dose) by intraperitoneal injection (*i.p.*) at 20 weeks after the final immunization. Nine days later, lymphocytes were isolated and stimulated with mixed antigens of ESAT-6, Ag85B, Rv2626c and HspX (2 μg/mL of each protein) for 4 h in vitro. Then, cells were incubated for 5–6 h with BD GolgiPlug™ (including brefeldin A, BD, USA) at 37 °C. Subsequently, cells were stained with anti-CD4-FITC (RM4-5, BD, USA) and anti-CD8-PerCP-Cy5.5 (53-6.7, BD, USA) at 4 °C for 30 min. Later, cells were permeabilized (Cytofix/Cytoperm kit, BD, USA) and intracellular cytokine (ICC) staining of anti-IFN-γ (XMG1.2, BD, USA) was performed at 4 °C for 30 min as previous reported [29]. All samples were run on ACEA NoveCyte.

### 2.9. TB10.4_4-12_ Pentamer Staining

Mice were treated with BCG (Danish 1331, 1 × 10^6^ CFU/dose) by *i.p.* After 9 days, lymphocytes were isolated and stained with TB10.4_4-12_ pentamer-PE (Pro5^®^ MHC Class I Pentamers, Pro Immune, Pro5^®^ MHC Class I Pentamers, Pro Immune) for TB10.4 specific CD8^+^ memory T cells at 22 °C for 10 min. Subsequently, cells were stained with anti-CD3-FITC (145-2C11, BD, USA) and anti-CD8-APC (53-6.7, BD, USA) at 4 °C for 30 min. Finally, samples were detected by flow cytometry.

### 2.10. BCG Challenge and Enumeration of Bacteria-Load

Mice were challenged with BCG (Danish 1331) at 1 × 10^7^ CFU/dose by intranasal route (*i.n.*). At 20 days later, bacterial colony forming units (CFU) in the lungs and spleens were detected. The homogenates of lungs and spleens were plated at 10-fold serial dilutions on Middlebrook 7H11 medium (BD, NJ, USA) and incubated at 37 °C for 3 weeks. Finally, CFU was enumerated.

### 2.11. Statistical Analysis

The results were presented as means ± SD. Comparisons were analyzed by one-way ANOVA and SPSS17.0 software. Data were considered as statistically significant at *p* < 0.05.

## 3. Results

### 3.1. rAd-IL-7-Linker-IL-15 Enhanced Quality and Quantity of T_CM_ Like Cells

**Figure 1:**
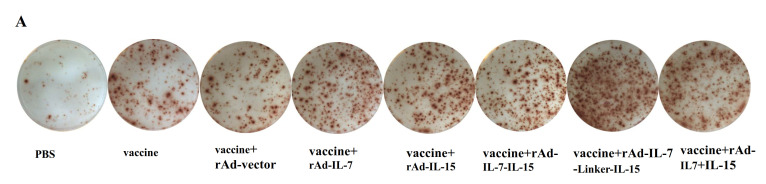

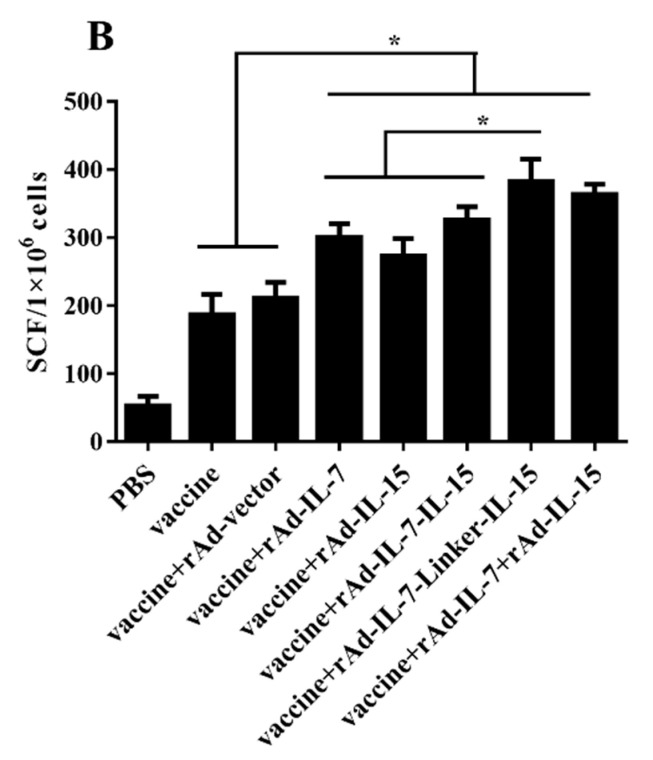
Cultured ELISPOT assay for antigen specific T_CM_ like cells. At 20 weeks after the last vaccination, lymphocytes of mice were cultured with or without a cocktail of antigens ESAT-6, Ag85B, Rv2626c and HspX for 9 days. Then, cells were re-stimulated with the same antigens for 20 h. (**A**) Representative images of IFN-γ ELISPOT wells from long-term cultured ELISPOT assays at 5× magnification. (**B**) Results of long-term cultured ELISPOT responses assay. Data were presented as means ± SD from groups of 4 mice. * *p* < 0.05.

To observe T_CM_ like cells-mediated immune responses induced by different cytokines, we performed cultured IFN-γ ELISPOT assay, IFN-γ secretion by ICC following twice-stimulation in vivo and in vitro, and the number of expanded TB10.4-specific CD8^+^ memory T cells at 20 weeks after the final immunization. In cultured ELISPOT assay, the results showed that the groups of vaccine + rAd-IL-7 (299.3 ± 20.9 SFC/1 × 10^6^ cells), vaccine + rAd-IL-15 (273.3 ± 25.2 SFC/1 × 10^6^ cells), vaccine + rAd-IL-7-IL-15 (325.6 ± 19.7 SFC/1 × 10^6^ cells), vaccine + rAd-IL-7-Linker-IL-15 (382.2 ± 32.9S FC/1 × 10^6^ cells) and vaccine + rAd-IL-7 + rAd-IL-15 (361.4 ± 17.2 SFC/1 × 10^6^ cells) induced a larger increase of long-term antigen-specific IFN-γ producing cells, which is the character of T_CM_ like cells [30], than the control groups of vaccine alone (186.0 ± 30.5 SFC/1 × 10^6^ cells) and vaccine + rAd-vector (211.6 ± 22.4 SFC/1 × 10^6^ cells). In the meantime, the group of vaccine + rAd-IL-7-Linker-IL-15 enhanced long-term antigen-specific IFN-γ producing cells, compared with the groups of vaccine + rAd-IL-7, and vaccine + rAd-IL-15 and vaccine + rAd-IL-7-IL-15. However, the group of vaccine + rAd-IL-7-Linker-IL-15 didn’t demonstrate an obvious difference with the group of vaccine + rAd-IL-7 + rAd-IL-15 (Figure 1).

**Figure 2:**
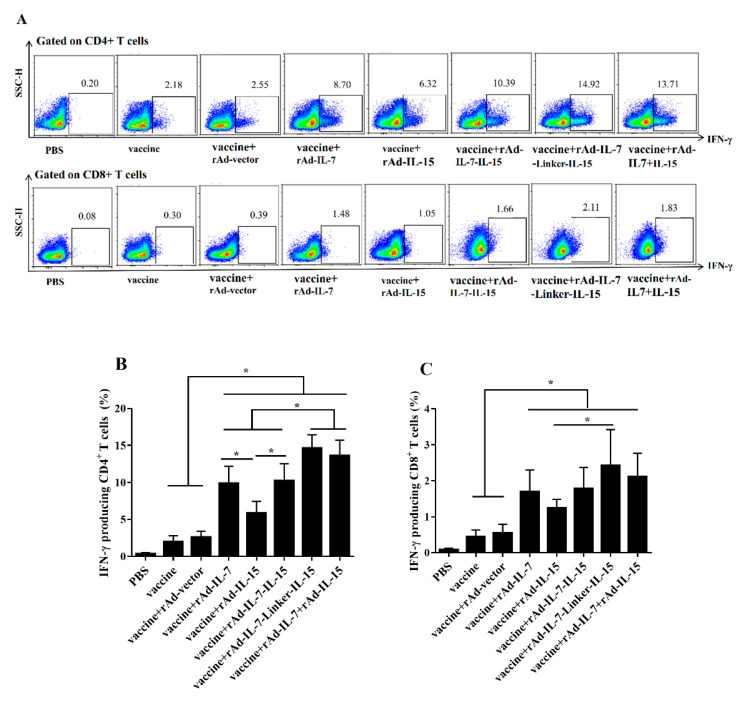
IFN-γ secretion following twice stimulation with antigens in spleens. At 20 weeks after the last immunization, mice were injected with BCG (1 × 10^6^ CFU) by *i.p* for 9 days. Then, lymphocytes of spleens were isolated and stimulated with a cocktail of antigens ESAT-6, Ag85B, Rv2626c and HspX (2 μg/mL) in vitro. Secretion of IFN-γ was determined by flow cytometry. (**A**) The representative results of every group; (**B**) CD4^+^ T cells secreting IFN-γ; (**C**) CD8^+^ T cells secreting IFN-γ. Data collected were presented as means ± SD from 5 mice per group. * *p* < 0.05.

Meanwhile, according to the principle of long-term cultured ELISPOT assay, we detected antigen specific T_CM_ like cells by IFN-γ secretion following twice-stimulation in vivo and in vitro sequentially [28]. Firstly, mice were injected with BCG by *i.p* at 20 weeks after the final immunization. Secondly, lymphocytes were isolated after 9 days later and stimulated for 4 h with mixed antigens in vitro. Then, ICC was performed. The data indicated that the vaccine + rAd-IL-7-Linker-IL-15 group induced higher frequency of IFN-γ on CD4^+^ T cells (14.64 ± 1.79%) than the groups of vaccine + rAd-IL-7 (9.87 ± 0.79%), vaccine + rAd-IL-15 (5.84 ± 1.62%), vaccine + rAd-IL-7-IL-15 (10.22 ± 2.34%), vaccine alone (2.02 ± 0.79%) and vaccine + rAd-vector control (2.63 ± 0.77%). The groups of vaccine + rAd-IL-7, vaccine + rAd-IL-15, vaccine + rAd-IL-7-IL-15 and vaccine + rAd-IL-7 + rAd-IL-15 produced higher levels of IFN-γ secretion than that of the control groups of vaccine alone and vaccine + rAd-vector. Moreover, the vaccine + rAd-IL-7 group had an increase of IFN-γ by 4.03% compared with the vaccine + rAd-IL-15 group (Figure 2B). For CD8^+^ T cells, the groups of vaccine + rAd-IL-7, vaccine + rAd-IL-15, vaccine + rAd-IL-7-IL-15, vaccine + rAd-IL-7-Linker-IL-15 and vaccine + rAd-IL-7 + rAd-IL-15 induced significantly more T_CM_ like cells immune responses compared to the control groups of vaccine and vaccine + rAd-vector. The frequency of CD8^+^ IFN-γ^+^ cells in the vaccine + rAd-IL-7-Linker-IL-15 group increased 1.18% compared with the group of vaccine + rAd-IL-15 (Figure 2C).

**Figure 3:**
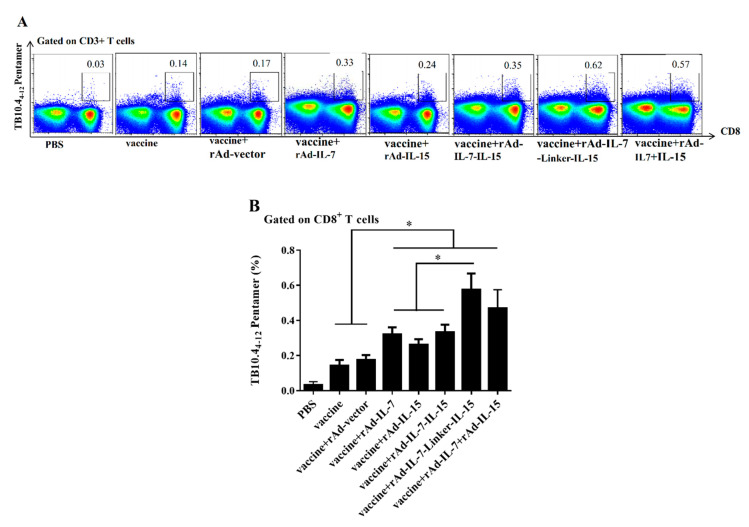
Number of expanded TB10.4-specific CD8^+^ T cells in spleens. Mice were injected with BCG by *i.p.* at 20 weeks after the final immunization. After 9 days, the number of TB10.4-specific CD8^+^ T_CM_ like cells was evaluated by TB10.4_4-12_ pentamer. (**A**) The representative results of every group; (**B**) Frequency percentages of TB10.4-specific CD8^+^ T_CM_ like cells. Data collected were presented as means ± SD from 5 mice per group. * *p* < 0.05.

Mice were injected with BCG by *i.p* at 20 weeks after the final immunization. After 9 days, the number of TB10.4-specific CD8^+^ memory T cells were evaluated by TB10.4_4-12_ pentamer, which was the same principle with IFN-γ secretion following twice-stimulation in vivo and in vitro. The results showed that the frequency of TB10.4-specific CD8^+^ T_CM_ like cells in the group of vaccine + rAd-IL-7-Linker-IL-15 was highest and had a significant increase compared with the groups of vaccine + rAd-IL-7, vaccine + rAd-IL-7-IL-15, vaccine + rAd-IL-15, vaccine + rAd-vector and vaccine. The groups of vaccine + rAd-IL-7, vaccine + rAd-IL-15 and vaccine + rAd-IL-7 + rAd-IL-15 induced more TB10.4-specific CD8^+^ T_CM_ like cells than the control groups of vaccine + rAd-vector and vaccine. There was no obvious difference between the groups of vaccine + rAd-IL-7-Linker-IL-15 and vaccine + rAd-IL-7 + rAd-IL-15 (Figure 3).

### 3.2. rAd-IL-7-Linker-IL-15 Induced Higher Proliferation Capability of T_CM_ Like Cells

**Figure 4:**
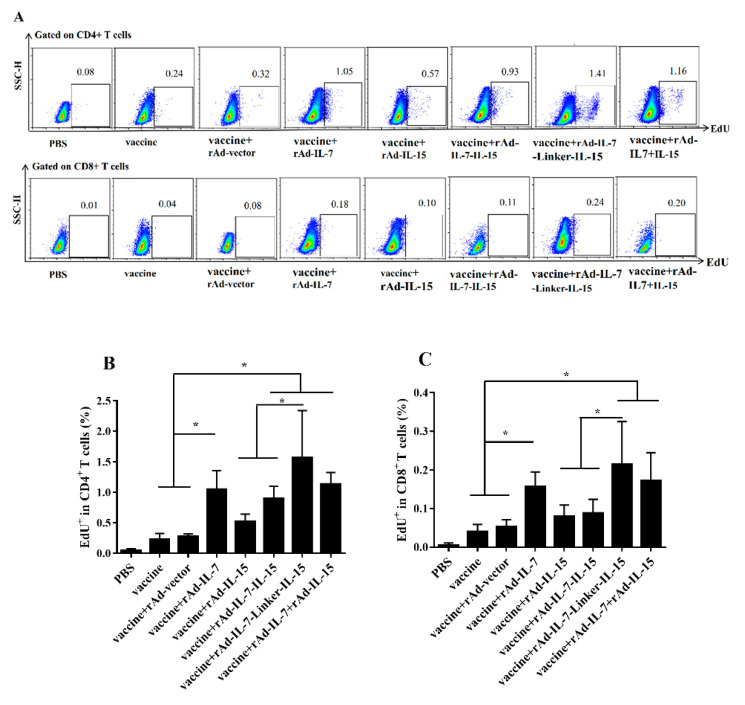
Proliferative capability of lymphocytes. For proliferation assay, 20 weeks after the last vaccination, lymphocytes (5 × 10^6^ cells/well) were stimulated with a cocktail of antigens ESAT-6, Ag85B, Rv2626c and HspX (2 μg/mL) for 7 days in 24-well plates. Three days after antigen stimulation, EdU was added at a final concentration of 30 μM and cells were cultured for another 4 days. At the 7th day, proliferative cells were detected by flow cytometry. (**A**) The representative results of every group. (**B**) Frequency percentages of EdU+ in CD4^+^ T cells; (**C**) Frequency percentages of EdU+ in CD8^+^ T cells. Results are presented as means ± SD from groups of 5 mice. * *p* < 0.05.

To evaluate proliferation of T_CM_ like cells induced by different cytokines, at 20 weeks after the last immunization, we performed EdU proliferation assay [28]. For CD4^+^ T cells, the groups of vaccine + rAd-IL-7, vaccine + rAd-IL-7-IL-15, vaccine + rAd-IL-7-Linker-IL-15 and vaccine + rAd-IL-7 + rAd-IL-15 showed an enhanced incorporation compared with the control groups of vaccine and vaccine + rAd-vector; The frequency of EdU^+^ T cells in the vaccine + rAd-IL-7-Linker-IL-15 group was highest, which was obviously higher than that of the vaccine + rAd-IL-15 group and vaccine + rAd-IL-7-IL-15; There was no significant difference among the groups of vaccine + rAd-IL-15, vaccine and vaccine + rAd-vector (Figure 4A,B). For CD8^+^ T cells, the tendency was consistent with CD4^+^ T cells, the groups of vaccine + rAd-IL-7, vaccine + rAd-IL-7-Linker-IL-15 and vaccine + rAd-IL-7 + rAd-IL-15 promoted the frequency of CD8^+^ T cells incorporated with EdU compares with that of the vaccine and vaccine + rAd-vector control groups (Figure 4A,C). In conclusion, the vaccine + rAd-IL-7-Linker-IL-15 group induced the strongest capability of proliferation on CD4^+^ T cells and CD8^+^ T cells among these groups. The group of the vaccine + rAd-IL-7 showed stronger capability of proliferation on CD4^+^ T cells and CD8^+^ T cells than the control groups of vaccine and vaccine + rAd-vector. The group of the vaccine + rAd-IL-15 showed no obvious difference compared with the control groups of vaccine and vaccine + rAd-vector.

### 3.3. rAd-IL-7-Linker-IL-15 Promoted the Protective Efficacy of Vaccine

**Figure 5:**
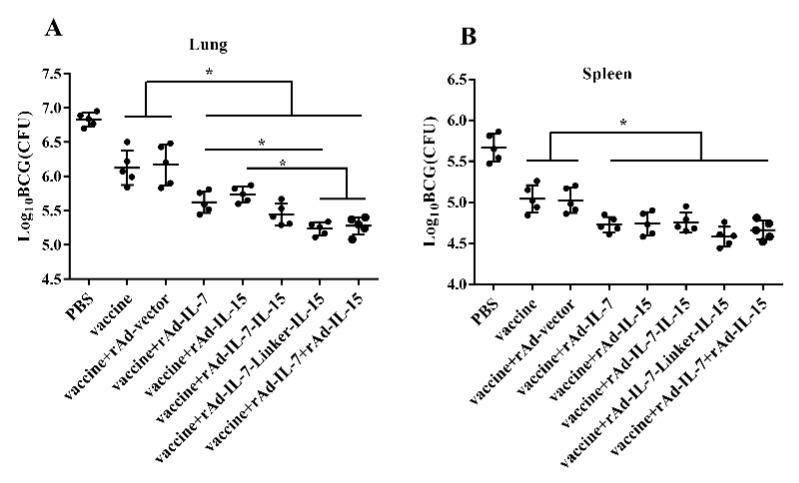
The protective efficacy of vaccines against BCG infection in mice. At 24 weeks after the last vaccination, mice were challenged with Mycobacterium bovis BCG 1 × 10^7^ CFU/100 μL/mice by nasal. 3 weeks post-challenge, (**A**) mice were euthanized and the bacterial burden (CFU) was measured in lungs; (**B**) mice were euthanized and the bacterial burden (CFU) was measured in spleens. Data are presented as log10 CFU ± SD from groups of 5 mice. * *p* < 0.05.

To identify the protective efficacy of vaccine associated with different cytokines, we examined CFU of the lungs and spleens after *Mycobacterium bovis* BCG challenge. At 24 weeks after the last immunization, mice were challenged with BCG. At three weeks post-challenge, CFU of the lungs and spleens was measured. Against BCG infection, the results showed that, CFU of the lungs in the groups of vaccine + rAd-IL-7, vaccine + rAd-IL-15, vaccine + rAd-IL-7-IL-15, vaccine + rAd-IL-7-Linker-IL-15 and vaccine + rAd-IL-7 + rAd-IL-15 all had a significant reduction compared with the control groups of vaccine and vaccine + rAd-vector; Moreover, the groups of vaccine + rAd-IL-7-Linker-IL-15 and vaccine + rAd-IL-7 + rAd-IL-15 had the least bacteria load, vaccine + rAd-IL-7-Linker-IL-15 declining approximately 0.38log10 CFU compared with the group of vaccine + rAd-IL-7 and 0.50log10 CFU compared with the group of vaccine + rAd-IL-15 (Figure 5A). In the spleen, the bacterial load in the groups of vaccine + rAd-IL-7 (4.73 ± 0.95log10 CFU), vaccine + rAd-IL-15 (4.74 ± 0.14log10 CFU), vaccine + rAd-IL-7-IL-15 (4.75 ± 0.12log10 CFU), vaccine + rAd-IL-7-Linker-IL-15 (4.58 ± 0.12log10 CFU) and vaccine + rAd-IL-7 + rAd-IL-15 (4.66 ± 0.12log10 CFU) were significantly lower than the control groups of vaccine (5.04 ± 0.17log10 CFU) and vaccine + rAd-vector (5.02 ± 0.15log10 CFU). However, there was no obvious difference among the groups of vaccine + rAd-IL-7-Linker-IL-15, vaccine + rAd-IL-7, vaccine + rAd-IL-15, vaccine + rAd-IL-7-IL-15 and vaccine + rAd-IL-7 + rAd-IL-15 (Figure 5B). These results indicated that IL-7-Linker-IL-15 promoted vaccine produce stronger immune memory with higher protective efficacy than IL-7 and IL-15.

## 4. Discussion

The ideal tuberculosis vaccines should be able to induce more T_CM_ to provide a long-term protection against TB. Currently, there are a few measures to prolong the protection of vaccines against TB. It is reported that low dose of antigen favors the induction of TCM while high dose of antigen mainly induces TEM or effective T cells [31,32]. It is also demonstrated that prolonging boosting intervals can induce a stronger booster response and enhanced long-term protective efficacy against *M. tuberculosis* [28,33]. Metformin could expand memory-like antigen-inexperienced CD8^+^ T cells and enhance protective efficacy against *M. tuberculosis* challenge [34]. Some studies have demonstrated that IL-7 and IL-15 can promote formation and homeostasis of memory T cells [35,36]. In our study, we explored T_CM_ like cells-mediated immunity induced by recombinant adenovirus encoding cytokines IL-7, IL-15, IL-7-IL-15, and IL-7-Linker-IL-15 combined with *Mycobacterium tuberculosis* subunit vaccine. We found that IL-7-Linker-IL-15 increased the quantity of T_CM_ like cells and enhanced proliferation capability compared with IL-7, IL-15, and IL-7-IL-15. Consistent with these, IL-7-Linker-IL-15 helped the vaccine produce higher protective efficacy against BCG than IL-7 and IL-15.

IL-7 plays crucial roles in both development of naïve T cells and expanding clonotypically diverse CD4^+^ and CD8^+^ memory T cells populations [12]. Our study showed that rAd-IL-7 promoted vaccine to induce more CD4^+^ T_CM_ like cells than the vaccine + rAd-IL-15 group, increasing the secretion of IFN-γ and proliferative capability following the repeated stimulation with same antigens in several days. For CD8^+^ T cells, the vaccine + rAd-IL-7 group increased T_CM_ like cells compared with the control groups of vaccine and vaccine + rAd-vector, with expanded number of TB10.4-specific CD8^+^ memory T cells and higher IFN-γ secretion following twice antigen stimulation, but the vaccine + rAd-IL-15 group didn’t. Taken together, this study showed that rAd-IL-7 resulted in significant increases in T_CM_ like cells compared with rAd-IL-15.

IL-15 plays a complicated effect on development of T_CM_ like cells, which may be related to the strength of IL-15 signaling. It is well-known that T cell development depends on IL-15β receptor, also known as CD122. Weak CD122 signaling supports T_CM_ development, while stronger CD122 signaling supports the development of T_EM_. Moreover, high CD122 signaling mainly promotes generation of short lived terminally differentiated effector T cells [37]. Our experiment showed that IL-15 have a weak effect on inducing T_CM_ like cells-mediated immune responses. On one hand, IL-15 contributes to maintaining the homeostasis of memory T cells [38,39]. On the other hand, IL-15 selectively promoted the proliferation of T_EM_ rather than T_CM_ [40].

It is interesting to point out that rAd-IL-7-Linker-IL-15, in which IL-7 and IL-15 is connected by a 12-amino acids linker (Gly-Gly-Gly-Ser)_3_, promote TB subunit vaccine to induce stronger long-term immune responses than rAd-IL-7-IL-15 and single rAd-IL-7 and rAd-IL-15. It has been demonstrated that this linker can minimize the refolding problems of the two fused chains, such as incorrect domain pairing or aggregation, and improves the stability of the structure. Consequently, it is beneficial for IL-7 and IL-15 to play a part in regulating the development of memory T cells [26,41,42]. Our study showed that rAd-IL-7-Linker-IL-15 promoted formation and maintenance T_CM_ like cells and improved proliferative capability of T_CM_ like cells, which resulted in stronger protective efficacy against BCG. Moreover, the results indicated that connection of IL -7 and IL-15 by the linker had a synergetic effect that had shown promising ability to produce unheralded biological effects to augment the T_CM_ like cells-mediated immune responses.

## 5. Conclusions

For the first time, our study demonstrates that supplementation of TB protein-subunit vaccine with rAd- IL-7-Linker-IL-15 would induce more T_CM_ like cells and improve its protective efficacy against *M. tuberculosis*. Meanwhile, IL-7 and IL-15 have been applied for the treatment of tumors in clinical trials and they were proved safe for patients [43,44,45]. However, IL-7 and IL-15 haven’t been used as adjuvants for the vaccine to healthy individuals in clinic. Therefore, fusion cytokine IL-7-Linker-IL-15 developed as an adjuvant need to be explored for triggering a stronger long-term cellular immune response against tuberculosis.

## Abbreviations

TB—Tuberculosis
*M. tuberculosis*—Mycobacterium tuberculosis
BCG—Mycobacterium bovis Bacilli Calmette-Guerin
TCM like cells—central memory-like T cells
TCM—central memory T cells
TEM—effector memory T cells
DDA—dioctadecylammonium bromide
Poly (I:C)—polyinosinic-polycytidylic acid
IL—Interleukin
LT70-ESAT6-Ag85B-MPT64<190-198>-Mtb8.4-Rv2626c
MH—Mtb10.4-HspX
PBS—Phosphate-buffered saline
rAd—recombined adenoviral
CPE—cytopathic effect
*i.p.*—intraperitoneal injection
APCs—antigen presenting cells
Linker—(Gly-Gly-Gly-Ser)_3_
